# Alcohol and Drink

**Published:** 1909-02-27

**Authors:** 


### ALCOHOL AND DRINK.

We are much interested in the controversy which has arisen between the brewery trade and scientific experts. We have long been alive to the fact that "all flesh is not the same flesh" is equally true with regard to alcohol. The question is not one of alcohol but one of drink. "What is the general effect on the human system of a beverage containing a very low percentage of alcohol combined with a large proportion of easily assimilable carbo-hydrates and proteins? What is the specific effect of such very diluted alcohol so blended with neutral matter and in such connection?" These are the questions put by Mr. Edwyn Barclay, the brewers' representative. They raise issues of great interest; and clear answers to the questions, apart from all prejudice, are of importance to the whole community. In pursuance of this idea The Hospital has devoted a considerable amount of time and expenditure to its investigations into the subject. Three years ago it published an exhaustive report of its Special Analytical Commission on the History, Manufacture, and Properties of Whisky and Distillery Spirits; two years ago it dealt with Light Wines in the same way; and it has now in the press, and will shortly publish, the report of its Special Analytical Commission on Beer. This note is written as we go to press, and we hope to deal with the matter at greater length in a leader next week.

